# A Multidimensional Study of Absorptive Capacity and Innovation Capacity and Their Impact on Business Performance

**DOI:** 10.3389/fpsyg.2021.751997

**Published:** 2021-10-27

**Authors:** Rafael Sancho-Zamora, Santiago Gutiérrez-Broncano, Felipe Hernández-Perlines, Isidro Peña-García

**Affiliations:** ^1^Department of Business Administration, Faculty of Law and Social Science, University of Castilla-La Mancha, Ciudad Real, Spain; ^2^Department of Business Administration, Faculty of Social Science, University of Castilla-La Mancha, Talavera de la Reina, Spain; ^3^Department of Business Administration, Faculty of Law and Social Science, University of Castilla-La Mancha, Toledo, Spain; ^4^Department of Business Administration, School of Computer Engineering, University of Castilla-La Mancha, Ciudad Real, Spain

**Keywords:** absorptive capacity, innovation capacity, organizational performance, potential absorptive capacity, realized absorptive capacity, product innovation, process innovation

## Abstract

The aim of this paper is to understand how absorptive capacity and innovativeness influence business performance. Most previous studies have not considered the different dimensions of absorptive capacity and innovativeness. As a consequence, they have not analyzed the relationships between these dimensions, such as potential and realized absorptive capacity (RACAP) and product and process innovation. In our study, we analyzed the relationships between each of these dimensions and their effect on organizational performance. To achieve this, in addition to the theoretical foundation provided by the working hypotheses, a questionnaire was sent to 800 CEOs of Spanish companies in different sectors, obtaining a response rate of 38.25%. Structural equation modeling was applied to test the hypotheses. This study confirms the positive effect of absorptive capacity on innovation capacity, which in turn has a positive effect on business performance. Moreover, different dimensions of absorptive capacity and innovativeness play an important role in these relationships. This study contributes to a better understanding of how potential and RACAP influence the innovativeness of firms, both in their ability to innovate products and to improve business processes. In addition, it explores how these different innovations impact business performance and provide firms with knowledge on how to invest resources to increase profits. Future research should further study the inner workings of each of the dimensions analyzed to determine the importance of each dimension for business performance.

## Introduction

In the knowledge economy era, innovation is a key source of competitive advantage (Daghfous, 2004; Prajogo and Ahmed, 2006). According to the knowledge-based vision, a firm’s performance is based on its ability to generate, combine, recombine, and exploit knowledge (Grant, 1996). Thus understood, knowledge is essential to a firm’s ability to innovate and compete, making it a strategic resource (Wang, 2013; Ibarra-Cisneros et al., 2021). A firm’s knowledge is usually produced through internal creation or external acquisition of information. Consequently, a firm’s knowledge absorptive capacity (AC) is important for value creation within the firm (Xie et al., 2018).

Davenport and Prusak (1998) assert that knowledge cannot be fully transferred without the support of absorptive capacity. Similarly, Szulanski (1996) reveals that knowledge transfer in a firm will emerge as a major obstacle without the support of absorptive capacity, placing value on the importance of absorptive capacity in firms (Wuryaningrat, 2013).

Absorptive capacity has been defined as “the ability of a firm to recognize the value of new external information, assimilate it and apply it for business purposes” (Cohen and Levinthal, 1990, p. 128) and has become one of the most prevalent research areas in business management (Huang et al., 2015). Zahra and George (2002) state that absorptive capacity is a set of organizational routines required to identify and utilize knowledge, highlighting the importance of absorptive capacity in the knowledge management process (Chang et al., 2012; Sancho-Zamora et al., 2021).

Many studies support the notion of absorptive capacity directly or indirectly influencing innovation and company financial results (i.e., Fosfuri and Tribó, 2008; Chen et al., 2009; Tseng et al., 2011). Processes of absorption of external knowledge have become essential elements for innovation in companies, enabling them to better adapt to changes in the competitive environment (Camisón and Forés, 2010). For this reason, there are still abundant research opportunities in the areas of relational learning, absorptive capacity, and the achievement of competitive advantage (Chen et al., 2009).

Xie et al. (2018) argue that two important gaps limit in-depth theoretical and empirical developments in absorptive capacity management. First, several studies have considered various dimensions of absorptive capacity (e.g., Camisón and Forés, 2010), although this dimensional division of the construct and its role is ambiguous, both in theory and practice. However, few studies have focused on the relationships between the multiple dimensions of absorptive capacity and firms’ innovation performance (e.g., Ahmed et al., 2020; Yaseen, 2020). Absorptive capacity is a tacit and complex construct, making it very difficult to measure. In this study, we adopt the two dimensions of Zahra and George (2002) to measure absorptive capacity, thus avoiding the use of a single index—such as R&D or R&D expenditure—to assess absorptive capacity (Liao and Wu, 2010).

Second, although several authors have suggested that each dimension of absorptive capacity plays distinct but complementary roles (Zahra and George, 2002; Najafi-Tavani et al., 2016; Flor et al., 2018), few studies have examined systematic theoretical and empirical testing of the internal mechanisms between the two dimensions of knowledge absorptive capacity.

In this paper, we mainly focus on bridging both gaps and analyzing the impact of different absorptive capacity dimensions on innovativeness. Furthermore, we differentiate between product innovation and process innovation, as suggested by some authors (Smith et al., 2005; Rush et al., 2007). We also study the effect of product innovation and process innovation on firm performance.

In order to test our hypotheses, empirical research was carried out on 315 Spanish companies, which served to validate our hypotheses and thus contribute to filling the existing gap in this field of research. Our research contributes to the existing literature by clarifying the role played by different dimensions of absorptive capacity in different types of innovation, and the effect of process and product innovation on business performance. Finally, alongside the conclusions, we present the limitations and business implications of this work. In addition, it presents different business implications, detailing the role that each of the dimensions of absorptive capacity plays in the development of innovations. The paper makes recommendations to facilitate the work of managers to focus their knowledge management if they intend to optimize innovations and achieve better economic results.

## Absorptive Capacity and Innovation

Firms are operating in a highly competitive environment and require high levels of knowledge, which has become one of their most valuable resources (Liao and Wu, 2010). In order to compete, firms cannot rely solely on their external knowledge network but also have to develop their absorptive capabilities to actively source knowledge (Matthyssens et al., 2005; Sancho-Zamora et al., 2021). This necessitates approaches and mechanisms that facilitate learning and thus enable them to disseminate and exploit the knowledge that will provide them with new organizational innovations (Daghfous, 2004). Moreover, the consolidation of this acquired knowledge is determined by the firm’s absorptive capacity (Sun and Anderson, 2010).

Firms therefore need to have, and to develop, internal absorptive capacity to improve their innovation performance. This is important because this type of capacity can influence the effectiveness of innovation activities (Cockburn and Henderson, 1998).

Cohen and Levinthal (1990) were the first to define absorptive capacity as a firm’s ability to evaluate new knowledge from outside, assimilate it, and apply it for commercial purposes (Wuryaningrat, 2013). It is a firm’s ability to acquire and effectively use external and internal knowledge that will subsequently affect their innovation (Daghfous, 2004; Fichman, 2004).

This approach views absorptive capacity as a by-product not only of R&D activities, but also of the diversity or breadth of the organization’s knowledge base, its prior learning experience, a shared language, the existence of cross-functional interfaces, and the mental models and problem-solving capacity of the organization’s members (Camisón and Forés, 2010). In this way, absorptive capacity is a critical factor for companies to use external knowledge and thus stimulate internal innovation (Dutse, 2013).

Knowledge has become the most important resource for firms; having external knowledge about markets and technologies is considered essential for the generation of internal knowledge in R&D departments (Cassiman and Veugelers, 2006). Through absorptive capacity, firms can transform external knowledge into innovation (Saebi and Foss, 2015). Initially, absorptive capacity starts with acquiring knowledge from the environment and it ends by exploiting it (Zahra and George, 2002; Jansen et al., 2006). This dynamic capacity allows firms to be in a better position to develop any kind of innovation (Andriopoulos and Lewis, 2009). Organizational learning theory suggests that a firm’s innovation performance is the result of its knowledge base (Griliches, 1990; Dodgson, 1993).

Previous research, such as that conducted by Schmidt and Rammer (2006), found that firms with higher absorptive capacity were more likely to carry out product, process, organizational, or even marketing innovations. Likewise, Calero-Medina and Noyons (2008) mapped studies related to absorptive capacity and its link to various domains, finding a significant relationship between absorptive capacity and organizational innovation. More recent work, such as Chen and Chang (2012), found that the higher the degree of absorptive capacity of the firm, the higher the degree of organizational innovativeness. Jantunen (2005) in his systematized review of the literature found that most existing research in the innovation literature emphasizes the importance of the ability to utilize external knowledge. Furthermore, this interaction with new external knowledge promotes absorptive capacity (Liao and Wu, 2010).

Research by Liao et al. (2007) provided empirical evidence that innovation results from the need for knowledge sharing, triggered by its absorptive capacity. When absorptive capacity improves, it becomes much easier for someone to create a remarkable innovation based on acquired knowledge. Indarti (2010) also mentions that absorptive capacity can be seen as a process through which a particular firm creates innovative business purposes (Wuryaningrat, 2013).

Despite all the existing evidence linking absorptive capacity to innovation, this concept has continued to develop over time. The most far-reaching reconceptualization was proposed by Zahra and George (2002). These authors linked the construct to a set of organizational routines and strategic processes through which firms acquire, assimilate, transform, and apply knowledge in order to create a dynamic organizational capability (Camisón and Forés, 2010).

### Dimensions of Absorptive Capacity

Zahra and George (2002) reformulated Cohen and Levinthal’s (1989) original three-dimensional model and elaborated a new one with four dimensions, which are grouped into two components: potential absorptive capacity (PACAP) and realized absorptive capacity (RACAP). Following these authors, we will consider absorptive capacity as a two-dimensional construct: While acquisition and assimilation represent the dimensions of PACAP, transformation and exploitation comprise the dimensions of RACAP (Müller et al., 2021).

Potential absorptive capacity focuses mainly on knowledge acquisition: on the one hand, the ability to value knowledge, as introduced by Cohen and Levinthal (1990) in relation to acquiring knowledge, and on the other hand, the ability to assimilate. Acquiring and using new information from the organization develops the breadth and depth of the firm’s existing knowledge base (Hu, 2014). A study conducted on manufacturing firms in different sectors established that close links with suppliers have a positive effect since suppliers bring new working methods to organizations (Porter and Heppelmann, 2015). Furthermore, the acquisition of new knowledge has been shown to have a positive relationship on manufacturing efficiency (West and Bogers, 2014) and the development of new value offerings (Phene et al., 2012). On the other hand, assimilating external knowledge involves incorporating it into routines and procedures for analyzing, processing, interpreting, and understanding information obtained from outside the organization. Knowledge assimilation represents its integration within organizational structures (Gebauer et al., 2012). Furthermore, information systems have been found to increase the importance of absorptive capacity for the success of innovation strategies (Kranz et al., 2016).

Realized absorptive capacity consists of the transformation and application of knowledge (Camisón and Forés, 2010). Transformation is considered as the ability to combine old and entrenched knowledge with newly acquired knowledge. This process takes place by adding new knowledge while re-evaluating and modernizing the organization’s old knowledge (Zahra and George, 2002). Considering the above, it can be deduced that by constructively combining old and new knowledge, original associations and links between different information flows emerge. This can lead to new perspectives on how to improve current activities or how to enter new markets in a differentiated way. While the former can lead to product innovation strategies, the latter can be considered market innovations or process innovations (Enkel et al., 2017). Finally, application refers to a firm’s ability to apply new external knowledge commercially to achieve organizational goals (Lane and Lubatkin, 1998); it involves both market and technological knowledge (Kranz et al., 2016). Market knowledge provides firms with information on how to commercialize their knowledge, while technological knowledge provides insights on how to develop new manufacturing methods (Teece, 2010). Thus, the desired outcome of absorptive capacity is the application of new knowledge for commercial purposes (Gebauer et al., 2012).

### Dimensions of Innovation Capacity

Innovation is a fundamental aspect of the research enterprise and is highly developed and present in all business processes (Chua et al., 1999; Alshanty and Emeagwali, 2019). However, the role of innovation as a key driver of business performance has changed in recent years due to globalization and increased international competition (Leal-Rodríguez and Albort-Morant, 2016; Pustovrh et al., 2017). We understand innovation as a firm’s ability to exploit knowledge and thereby generate new products, services, and processes (McDowell et al., 2018). However, innovation always involves a certain amount of risk, which is why the results are not always satisfactory (Hernández-Perlines et al., 2020).

Different studies have shown that innovativeness enables firms to achieve results, such as: improving firm performance (Jiménez-Jiménez and Sanz-Valle, 2011); increasing exports (Love and Roper, 2015); generating a competitive advantage (Coccia, 2017); and/or contributing to business growth (George et al., 2012). Overall, innovation helps firms respond to competitive challenges in globalized environments (Hausman and Johnston, 2014).

In this research, innovativeness is understood as an outcome of both potential and RACAP (Zahra and George, 2002; Winter, 2003). But it is a very complex ability in which new knowledge and ideas are continuously applied with the aim of achieving business performance through the incorporation of new offerings—product innovation—and the development of new procedures for making and distributing those offerings—process innovation (Smith et al., 2005; Rush et al., 2007), thus increasing or maintaining their effectiveness and competitiveness. Specifically, following Liao et al. (2007) and Damanpour and Gopalakrishnan (2001), we define two dimensions of innovativeness that include process innovation and product innovation. Process innovation focuses on improving the efficiency and internal workings of the firm’s processes to manufacture, assemble, or deliver the product. In this way, a new process can reduce costs or generate more production capacity for the company. Product innovation, on the other hand, is where a company can bring better, differentiated, improved, or even new products to the market to meet customer needs. Product innovation focuses on the market and relies on strong capabilities, such as quality, efficiency, speed, and flexibility (Lawson and Samson, 2001), while process innovation belongs to the realm of technical innovation (Liao et al., 2007). Both types of innovation are very closely linked and constitute complex processes that usually involve all functional areas of the company (Fores and Camisón, 2011).

In view of the above, the relationship between absorptive capacity and innovation capacity is supported by the literature. Likewise, we find sufficient grounds to identify different dimensions for both absorptive capacity and business innovations. Therefore, we propose the following hypotheses:

*H1*: PACAP influences (+) product innovation (PROTINN).

*H2*: RACAP influences (+) product innovation (PROTINN).

*H3*: PACAP influences (+) process innovation (PROCINN).

*H4*: RACAP influences (+) process innovation (PROCINN).

According to Zahra and George (2002), both ACAP and RACAP play separate but complementary roles. Firms cannot apply external knowledge without first acquiring it. Similarly, some organizations can develop, acquire, and assimilate external knowledge but are sometimes unable to transform and apply this knowledge, i.e., to turn it into innovations and thus into competitive advantage. Therefore, both subsets of ACAP fulfill a necessary but not sufficient condition to generate value in the company through the innovations implemented (Camisón and Forés, 2010). Thus, we establish the following hypothesis:

*H5*: The PACAP influences (+) the RACAP.

## Innovation and Performance

The generation and adoption of innovation enable firms to adapt to changes in the environment and to achieve their objectives. This is especially important in conditions of intense competition, where customers are better informed and demand increasingly higher-quality products and services (Jansen et al., 2006; Damanpour et al., 2009; Fernández and Peña, 2009). The development of an innovation strategy requires a combination of the firm’s internal learning and absorptive capabilities (Fores and Camisón, 2011). There is a general consensus that innovation is a strong competitive advantage; numerous studies link innovation with improved business performance (Leal-Rodríguez and Albort-Morant, 2016).

Chen et al. (2009), in addition to finding a direct relationship between absorptive capacity and innovativeness, showed that improved innovativeness has a positive impact on business performance. Moreover, Camisón and Villar-López (2014) found from a sample of 144 Spanish firms that organizational innovation favors the development of technological innovation competences and that both can contribute to improved firm performance.

Exposito and Sanchis-Llopis (2018), using a large sample of Spanish SMEs, highlighted the positive impact of innovation on different performance indicators: increase in sales, cost reduction, increase in productive capacity, and cost improvement. Furthermore, they proposed analyzing the relationship between innovation and business performance from a multidimensional analytical approach, as different types of innovation have a different impact depending on the outcome indicator considered.

Based on the previous literature, and from the multidimensional approach recommended by Exposito and Sanchis-Llopis (2018), we formulate the following hypotheses:

*H6*: Product innovation (PROTINN) influences (+) business performance (PERF).

*H7*: Process innovation (PROCINN) influences (+) business performance (PERF).

## Methodology

### Data Collection

Data were obtained from a questionnaire mailed to 800 randomly selected small and medium-sized enterprises in the Spanish autonomous community of Castilla-La Mancha. Contacts for the questionnaire were obtained from the SABI database, and active enterprises belonging to different sectors of activity in both the industrial and service sectors were selected. A total of 315 questionnaires were obtained, of which nine were rejected as incomplete (see Table 1).

**Table 1:** Research technical data.

Sample size	15,853 companies 800 randomly selected
Unit of analysis	Company
Scope	Castilla-La Mancha (Spain)
Valid responses/Response rate	306/38.25%
Confidence level	95%
Error rate	5.55%
Informant	CEOs
Data	October–December 2019

Table 2 shows the sectors and the activity of the participating companies.

**Table 2:** Sector and activity of the analyzed companies.

Sectors (CNAE)	Code	Activity	Number	Percentage
62, 69, 70, 71, 73	1	Specialized consulting services	75	24.50%
41, 43	3	Construction	65	21.24%
55, 56, 46, 47, 68	2	Retail and accommodation services	96	31.37%
10, 11, 14, 18, 21, 23, 25, 26, 27, 28, 31	4	Manufacturing	70	22.87%

The statistical power of the sample used in this study was 0.998 and was calculated using Cohen’s (1992) retrospective test, which can be obtained with the program G * Power 3.1.9.2 (Faul et al., 2009). The value obtained allows us to affirm that the sample used in this study has adequate statistical power as it is above the threshold of 0.80 established by Cohen (1992).

### Measurement of the Variables

All variables were measured using a 7-point Likert scale ranging from 1 (strongly disagree) to 7 (strongly agree). Specifically, the following variables were used in this study (see Table 3):

a) Measurement of PACAP. PACAP was operationalized as a second-order composite type A, based on acquisition capacity (three items) and assimilation capacity (four items). The scales proposed by Cohen and Levinthal (1990) and Lane et al. (2006) were used for its measurement. This scale has been validated by Flatten et al. (2011) and Hernández-Perlines et al. (2016).
b) Measurement of RACAP. RACAP was operationalized as a second-order composite type A, based on transformation capacity (four items) and exploitation capacity (three items). The scales proposed by Cohen and Levinthal (1990) and Lane et al. (2006) were used for its measurement. This scale has been validated by Flatten et al. (2011) and Hernández-Perlines et al. (2016).
c) Measurement of product innovation. Product innovation was operationalized as a first-order composite type A, with five items from the scale proposed by Prajogo and Sohal (2006). This scale has been validated in previous studies, such as Hernández-Perlines et al. (2019).
d) Measurement of process innovation. Product innovation was operationalized as a first-order composite type A, with four items from the scale proposed by Prajogo and Sohal (2006). This scale has been validated in previous studies, such as Hernández-Perlines et al. (2019).
e) Performance measurement. To measure performance, we have used an overall measure of firm performance that assesses the perception of firm performance relative to its competitors (Olson et al., 2005). The use of perception or satisfaction measures as determinants of firm performance is increasingly common in research (Manzano-García and Ayala-Calvo, 2020). Performance was operationalized as a first-order composite type A. The four items used in this research were as: sales growth, profit growth, market share growth, and return on equity growth. All of them have been extracted from a combination of the scales proposed by Chirico et al. (2011); Kellermanns et al. (2012); Krauss et al. (2005); Naldi et al. (2007); and Wiklund and Shepherd (2003). This scale has been validated by Hernández-Perlines et al. (2021).
f) Control variables. In this research, size (number of employees) and seniority (number of years since incorporation), as proposed by Chrisman et al. (2005) and validated by Ibarra-Cisneros and Hernández-Perlines (2020), were used as control variables. All control variables were operationalized as first-order composites type A.

**Table 3:** Measurement of variables.

Variables	Manner of operationalization	Number of items	Authors
Potential Absorptive Capacity (PACAP)	Second-order composite type A	7	Cohen and Levinthal (1990); Lane et al. (2006)
Realized Absorptive Capacity (RAPAC)	Second-order composite type A	7	Cohen and Levinthal (1990); Lane et al. (2006)
Product Innovation (PRODINN)	First-order composite type A	5	Prajogo and Sohal (2006)
Process Innovation (PROCINN)	First-order composite type A	4	Prajogo and Sohal (2006)
Performance (PERF)	First-order composite type A	4	Chirico et al. (2011); Kellermanns et al. (2012); Krauss et al. (2005); Naldi et al. (2007); Wiklund and Shepherd (2003)

## Results

To analyze the results and test both the direct and moderating hypotheses proposed in this paper, the multivariate partial least squares (PLS) quantitative structural equation technique was employed.

The choice of this method of data analysis is justified for the following reasons:

a) It is an appropriate method of analysis when research is in the early stages of developing new theoretical constructs (Gefen et al., 2011; Ringle et al., 2015).
b) It is a method of analysis characterized by its predictive nature, which makes it possible to address the research questions posed (Hair et al., 2014; Sarstedt et al., 2014).
c) Through this method of analysis, it is possible to observe the different causal relationships between the variables analyzed (Jöreskog and Wold, 1982; Astrachan and Jaskiewicz, 2008).
d) It is a suitable method of data analysis when the sample is not very large (Reinartz et al., 2009; Henseler et al., 2015).
e) It is a method that allows the analysis of complex model relationships (Hair et al., 2019).

The software used for data analysis using SEM-PLS was SmartPLS v.3.3.3 (Ringle et al., 2015).

To analyze the results, the recommendations of Barclay et al. (1995) and Hair et al. (2017) were followed, which advise first evaluating the measurement model and then evaluating the structural model.

To follow the evaluation process of both the measurement and structural models, the variables were modeled following the method described by Sarstedt et al. (2016) in order to analyze them with PLS:

a) The PACAP was operationalized as a second-order compound type A.
b) Realized absorptive capacity was operationalized as a second-order compound type A.
Product innovation was operationalized as a first-order composite type A.
c) Process innovation was operationalized as a first-order composite type A.
d) Performance was operationalized as a first-order composite type A.
e) The three control variables (age, sector, and size) were operationalized as a first-order composite type A.

To evaluate the measurement model, the variables were checked for reliability and adequate levels of convergent and discriminant validity, following the recommendations of Roldán and Sánchez-Franco (2012). For this purpose, the following indicators were used (Barclay et al., 1995; Roldán and Sánchez-Franco, 2012; Hair et al., 2017):

a) Composite reliability should have values above 0.7 according to Fornell and Larcker (1981), with appropriate values being those between 0.7 and 0.9 (Hair et al., 2018). All model indicators have acceptable composite reliability values (see Table 4). Furthermore, the composite reliability does not present redundancy problems because no value is higher than 0.95 (Drolet and Morrison, 2001; Diamantopoulos et al., 2012).
b) Cronbach’s Alpha values above 0.7 (Fornell and Larcker, 1981). In our case, Cronbach’s Alpha is higher than this value for all variables (see Table 4).
c) The Rho a must be greater than 0.7 (Dijkstra and Henseler, 2015) and must lie between the values of composite reliability and Cronbach’s Alpha (Hair et al., 2018). This condition is met for the different variables (see Table 4).
d) Average variance extracted (AVE) can be used to assess the convergent validity of each composite. Fornell and Larcker (1981) recommend a value higher than 0.5 for the AVE. This condition is valid for our data (see Table 4).
e) Heterotrait-Monotrait ratio (HTMT) allows us to measure discriminant validity. It is necessary to check that the correlation between each pair of constructs is not greater than the square root value of the AVE of each construct. For discriminant validity to hold, HTMT values must be less than 0.85 (Henseler et al., 2015). Discriminant validity is confirmed when the indicated values are met (see Table 4).

**Table 4:** Correlation matrix, composite reliability, convergent and discriminant validity, Heterotrait-Monotrait ratio (HTMT), and descriptive statistics.

Construct	AVE	Composite reliability	PACAP	RACAP	PROTINN	PROCINN	PERF
1. PACAP	0.893	0.943	0.944^*^				
2. RACAP	0.900	0.947	0.764	0.948^*^			
3. Product innovation (PROTINN)	0.618	0.889	0.677	0.726	0.786^*^		
4. Process innovation (PROCINN)	0.652	0.881	0.605	0.607	0.767	0.807^*^	
5. Performance (PERF)	0.722	0.912	0.273	0.177	0.206	168	0.846^*^
**Heterotrait-Monotrait rate (HTMT)**		
1. PACAP							
2. RACAP			0.584				
3. Product innovation (PROTINN)			0.760	0.812			
4. Process innovation (PROCINN)			0.694	0.703	0.653		
5. Performance (PERF)			0.0.264	0.192	0.171	0.187	
Cronbach’s Alpha			0.880	0.888	0.846	0.821	0.875
Rho A			0.888	0.890	0.871	0.856	0.900
Mean			4.09	4.35	4.02	4.38	3.96
SD			1.12	1.31	1.19	0.98	0.99

* *The values of the diagonal were obtained from the square root of the AVE of each compound*.

*The mean and standard deviation values of each of the second-order composites were calculated from the mean values of the different first-order composites of which they are composed*.

To complete the verification of discriminant validity, we also computed the HTMT inference from the bootstrapping option (5,000 subsamples). When the resulting interval contains values less than 1, discriminant validity exists, and our data meet this requirement (see Table 5).

**Table 5:** HTMT inference.

	Original sample (O)	Sample mean (*M*)	5.0%	95.0%	Sample mean (*M*)	Bias	5.0%	95.0%
PACAP- > RACAP	0.864	0.863	0.827	0.893	0.863	0.001	0.824	0.891
PACAP- > PROTINN	0.197	0.203	0.031	0.372	0.203	0.007	0.020	0.359
RACAP- > PROTINN	0.556	0.551	0.383	0.710	0.551	−0.005	0.389	0.714
PACAP- > PROCINN	0.197	0.203	0.031	0.372	0.203	0.006	0.02	0.359
PACAP- > PROCINN	0.318	0.321	0.079	0.553	0.321	0.003	0.068	0.545
PROTINN- > PERF	0.064	0.072	−0.194	0.350	0.072	0.008	−0.202	0.342
PROCINN- > PERF	0.150	0.152	−0.215	0.451	0.152	0.002	−0.281	0.411

**Figure 1:**
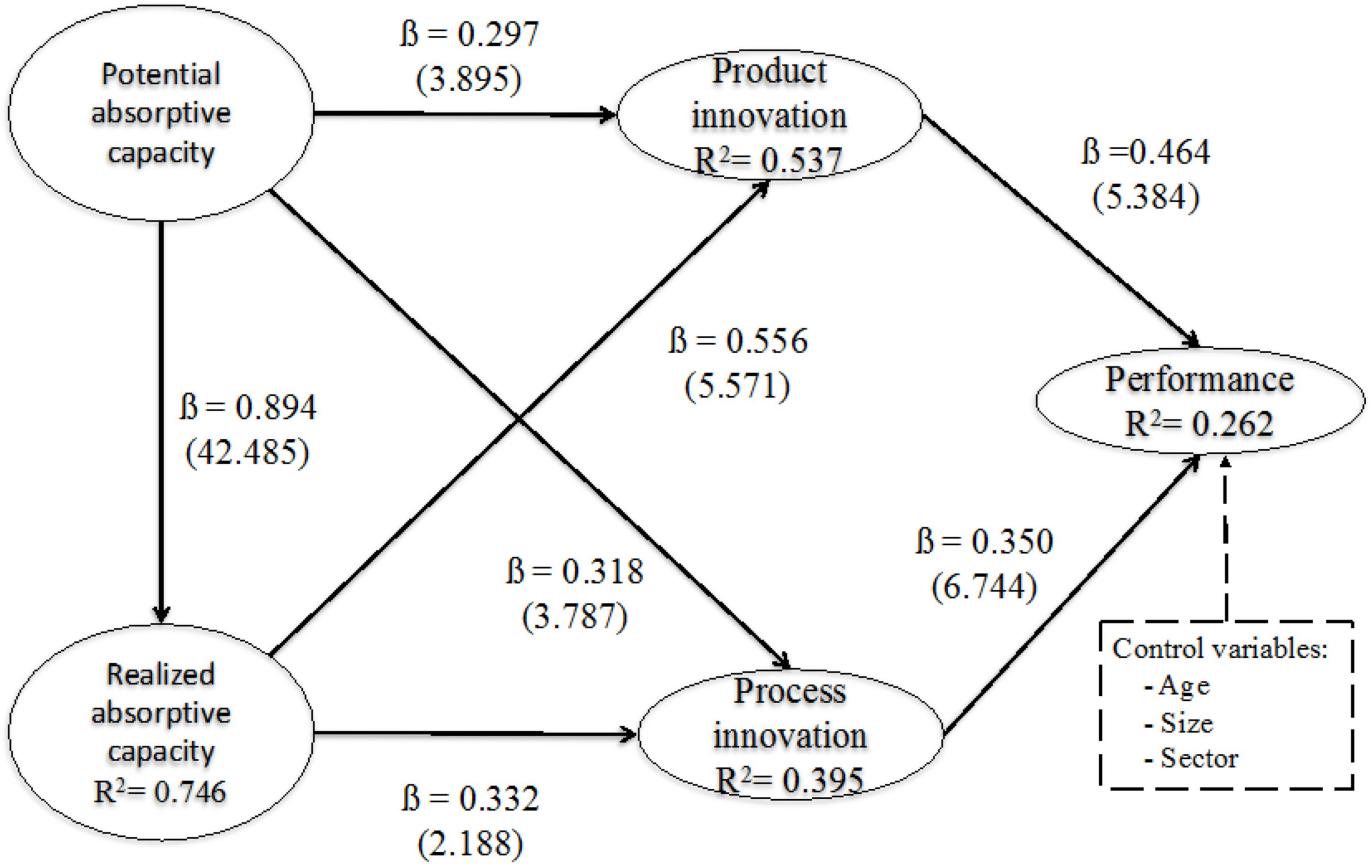
Structural model.

Having confirmed the convergent and discriminant validity of the measurement model, we proceeded to check the relationships between the different variables in order to carry out a structural model analysis. The analysis of the structural model will be discussed according to the relationships proposed in the research model (see Table 6 and Figure 1).

**Table 6:** Structural model.

Relations	*ß*	*t*-value	Hypothesis
PACAP > PROTINN	0.297	3.895	*H_1_: Supported*
RACAP > PROTINN	0.556	5.571	*H_2_: Supported*
PACAP > PROCINN	0.318	3.787	*H_3_: Supported*
RACAP > PROCINN	0.332	2.188	*H_4_: Supported*
PACAP > RACAP	0.894	42.485	*H_5_: Supported*
PROTINN > PERF	0.464	5.384	*H_6_: Supported*
PROCINN > PERF	0.350	6.744	*H_7_: Supported*

- First of all, the model suggests a positive and significant relationship between PACAP and product innovation (path coefficient=0.297; *t*-value=3.895). This influence is positive, as the path coefficient is positive and higher than 0.1. These results confirm the first hypothesis.

- Second, the model suggests a positive and significant relationship between RACAP and product innovation (path coefficient=0.556; *t*-value=5.571). These results confirm the second hypothesis.

- Third, the model suggests a positive and significant relationship between PACAP and process innovation (path coefficient=0.318; *t*-value=3.787). These results confirm the third hypothesis.

- Fourth, the model suggests a positive and significant relationship between RACAP and process innovation thesized. (path coefficient=0.332; *t*-value=2.188). These results confirm the fourth hypothesis.

- Fifth, the model suggests a positive and significant relationship between the PACAP and the RACAP (path coefficient=0.864; *t*-value=42.485). These results confirm the fifth hypothesis.

- Sixth, the model suggests a positive and significant relationship between product innovation and performance (path coefficient=0.464; *t*-value=5.384). These results confirm the sixth hypothesis.

- Seventh, finally, the model suggests a positive and significant relationship between process innovation and performance (path coefficient=0.350; *t*-value=6.744). These results confirm the seventh hypothesis.

It is also important to check the percentage explanation of the variance of the dependent variables. In this sense, the model proposed is capable of explaining 74.6% of the variance of RACAP from the PACAP (see Table 7 and Figure 1). The variance of product innovation is explained by the PACAP and RACAP, accounting for 53.7% of the variance (see Table 7 and Figure 1). The variance of process innovation is explained by PACAP and RACAP to the extent of 39.5% (see Table 7 and Figure 1). Finally, performance is explained by product innovation and process innovation, so that both types of innovation explain 26.2% of the variance of performance (see Table 7 and Figure 1). If we look at the different paths and the path coefficients, we can define the most appropriate route to improve performance based on absorptive capacity and innovation. As shown in Figure 1, the PACAP is an antecedent of the RACAP (*B*=0.894). RACAP is an antecedent of product innovation (*B*=0.556) and product innovation is an antecedent of performance (0.464). Therefore, the best way to achieve performance is through PACAP, RACAP, and product innovation.

**Table 7:** Explanation of variance.

Variable	*R* ^2^
*RACAP*	74.6
*Product Innovation*	53.7
*Process Innovation*	39.5
*Performance*	26.2

None of the control variables have an influence that can be considered relevant (path coefficients are less than 0.2), and they are not significant (their value is less than the recommended value, *p*<0.001; see Table 8).

**Table 8:** Control variables.

Variable	*ß*	*t*-valor
Age	−0.097	0.982
Sector	−0.089	0.679
Size	0.071	0.551

To complete the analysis of the structural model, the goodness of fit of the model was calculated through the standardized root mean square residual (SRMR) proposed by Hu and Bentler (1998) and Henseler et al. (2015). The SRMR value is 0.069 (lower than the value of 0.08 recommended by Henseler et al., 2015) as adequate.

## Discussion

Drawing on the most recent literature on dynamic capabilities, this study conducted an empirical analysis to demonstrate the impact of different dimensions of absorptive capacity on different types of innovation (H1–H4), product innovation, and process innovation, as suggested by some authors (Smith et al., 2005; Rush et al., 2007). Only a few studies have focused on the relationships between the multiple dimensions of absorptive capacity, innovativeness, and business performance.

Second, we tested the positive impact of the different types of innovation proposed on business performance (H6 and H7). The results obtained are consistent with previous theoretical and empirical literature relating ACAP (Limaj et al., 2016) and innovation to business performance (Fernández and Peña, 2009).

Furthermore, a positive and significant relationship was found between PACAP and RACAP (H5). This research addresses a gap in the literature regarding the direct and positive relationship between PACAP, RACAP, and firms’ innovation, in line with Yaseen’s (2020) proposal. Potential and RACAP represent different but complementary roles, because knowledge cannot be transformed and exploited if it has not been previously acquired and assimilated. This suggests that acquiring absorptive capacity is a sequential process that allows outside knowledge to be absorbed, recognizing its value, and proceeding to understand and combine it with internal knowledge in order to subsequently generate new knowledge. These results are in line with the proposal of Zahra and George (2002), since PACP allows competitive advantage in innovation to be achieved but will be superior when firms develop their capacity to transform and exploit external knowledge (RACAP).

For companies committed to the acquisition and assimilation of external knowledge, and the development and refinement of routines that facilitate combining existing and newly acquired knowledge, better product and process innovation results are achieved, which has an impact on business performance. In this way, we can affirm that companies with greater absorptive capacity make much better use of all the information captured from external sources and improve their results. In rapidly changing environments, this is essential for the improvement of their processes and products to improve their competitive position. The theoretical literature on ACAP postulates that greater investment in knowledge creation increases absorptive capacity, which ultimately helps firms to achieve higher innovative and financial performance.

This paper contributes to the literature on absorptive capacity and innovation management and provides several insights for practitioners, highlighting the importance of transforming and exploiting acquired knowledge to improve innovation capacity and overall business performance. Competitiveness requires an organizational culture that fosters knowledge acquisition and learning. Thus, companies must focus on retaining and recruiting employees with prior knowledge related to experience to take advantage of the knowledge generated. From our point of view, skilled personnel are at the core of absorptive capacity since they are the ones who can value, assimilate, transform, and exploit knowledge and produce innovation. Since knowledge resides in the people that make up a company, organizational absorptive capacity is more than the sum of individual capacities; therefore, companies must create communication structures and internal information flows to favor the innovation process. As a way of accessing external knowledge, companies should build cooperation networks with other companies that favor innovation and encourage the geographical and organizational mobility of qualified personnel.

The results of this study should be viewed and interpreted with some caution due to several limitations. One of the limitations of the study relates to the use of cross-sectional data, which does not enable exact causal relationships to be established. Second, respondents provided us with information on absorptive and innovation capacity and business performance. In this situation, there is a tendency for respondents to more positively rate those variables over which they have a more direct influence, and in some cases, they may not have exact knowledge about certain performance indicators. In this paper, we have seen how PACAP influences RACAP, thus supporting Zahra and George’s (2002) proposal that the two dimensions are considered distinct but complementary. However, these dimensions can also act separately, as established through a systematic theory, and therefore, we recommend a stronger analysis of the inner workings between the different dimensions of absorptive capacity. Future lines of research should be aimed at overcoming the aforementioned limitations and broadening the scope of the study as a consequence of the findings obtained in this research, in terms of other possible contingencies that condition the relationships set out in the paper.

## Data Availability Statement

The raw data supporting the conclusions of this article will be made available by the authors, without undue reservation.

## Author Contributions

All authors listed have made a substantial, direct, and intellectual contribution to the work, and approved it for publication.

## Funding

The publication of this article was financed by the Faculty of Law and Social Sciences of Ciudad Real, University of Castilla-La Mancha.

## Acknowledgments

Thank you to small and medium-sized enterprises in the Spanish autonomous community of Castilla-La Mancha for their support of this research.

## Conflict of Interest

The authors declare that the research was conducted in the absence of any commercial or financial relationships that could be construed as a potential conflict of interest.

## Publisher’s Note

All claims expressed in this article are solely those of the authors and do not necessarily represent those of their affiliated organizations, or those of the publisher, the editors and the reviewers. Any product that may be evaluated in this article, or claim that may be made by its manufacturer, is not guaranteed or endorsed by the publisher.
